# Alternative to Animal Use for Detecting Biologically Active Staphylococcal Enterotoxin Type A

**DOI:** 10.3390/toxins10120540

**Published:** 2018-12-15

**Authors:** Reuven Rasooly, Paula Do, Xiaohua He, Bradley Hernlem

**Affiliations:** Western Regional Research Center, Foodborne Toxin Detection & Prevention Research Unit, Agricultural Research Service, United States Department of Agriculture, Albany, CA 94710, USA; paula.do@ars.usda.gov (P.D.); Xiaohua.He@ars.usda.gov (X.H.); bradley.hernlem@ars.usda.gov (B.H.)

**Keywords:** staphylococcal enterotoxin type A, T-cell line, B-cell line, splenocyte

## Abstract

Staphylococcal enterotoxins (SEs) are a food safety concern. Existing methods for biologically active SE detection rely on the emetic response in live kittens or monkeys. This method suffers from low sensitivity, poor reproducibility, and causes ethical concerns regarding the use of experimental animals. The Lautenberg Chemical Safety Act encourages the development and adoption of alternatives to testing on animals for chemical toxicity methodologies. In this study, we utilized the superantigenic effect of SE type A (SEA) and used an ex vivo bioassay as an alternative to live animal testing. We found that interleukin-2 (IL-2) secreted by splenocyte can be utilized for quantifiable detection of SEA in food products. To avoid food matrix interference and attenuation of signal, we separated SEA from spiked food products by employing immunomagnetic beads that were coated with an anti-SEA antibody. This ex vivo method has achieved the detection of 1 ng mL^−1^ of SEA, which is 10^7^ times more sensitive than the existing live animal testing methods. However, this ex vivo bioassay requires sacrificing of mice. To overcome this limitation, we established a cell based in vitro assay using CCRF-CEM, a human CD4^+^ T-cell line, for the quantitative detection of SEA. Incubation of SEA with CCRF-CEM human T-cells and Raji cells led to quantifiable and dose dependent secretion of IL-2. This novel cell-based assay is highly specific to biologically active SEA, compared with the related SE toxin subtypes B, D, and E or heat inactivated SEA, which produce no secretion of IL-2. This is the first demonstration of an alternative assay that completely eliminates the use of animals for quantitative detection of active SEA.

## 1. Introduction

*Staphylococcus aureus* is a prevalent bacterial pathogen causing foodborne diseases that affect about a quarter million people every year in the United States (US) [1]. Pathogenesis is brought about, most notably, by virulence factors comprising some twenty-three different staphylococcal enterotoxins (SEs) produced by *S. aureus* and which are involved in food poisoning. These toxins affect the gastrointestinal tract, induce emesis, and activate the immune system by binding to the major histocompatibility complex (MHC) class II molecules of antigen presenting cells (APC). They also bind to the variable region (Vβ) on the T-cell receptor forming a bridge between APC and T-cells, causing activation of CD4^+^ T-cells. The absence of T-cell activation has been shown to be associated with the absence of emetic response [2,3,4].

Staphylococcal enterotoxin A (SEA) is regarded as the most accepted causative agent of foodborne disease outbreaks worldwide. For instance, SEA contamination of milk produced an extensive outbreak affecting as many as 13,420 people in Japan [5,6]. A complete study of 359 outbreaks that developed in the United Kingdom revealed that 79% of the identified *S. aureus* strains produced SEA [7]. Furthermore, SEA was the most prevalent enterotoxin recovered from food poisoning outbreaks in the US (77.8% of all outbreaks) [8]. These outbreaks emphasize the necessity to develop improved methods to detect active SEA, so as to prevent them from entering the human food chain. 

The presently accepted test to detect biologically active SEA is an in vivo monkey or kitten bioassay [9,10]. This method suffers from poor reproducibility, low sensitivity, and also raises ethical concerns regarding the use of experimental animals. Immunological methods, e.g., ELISA, latex agglutination, and mass spectrometry have been developed for several SEs [10,11,12]. They are unable to differentiate between active and inactive SEA. Also, antibodies against SEA can react with food matrices to give false-positive results [13]. Cell-based toxin active assays have been developed for detection and quantification of the related Staphylococcal enterotoxin type E (SEE) [14,15]. Jurkat T-cells were used in those assays for SEE and secretion of the cytokine interleukin-2 (IL-2) by Jurkat cells were used for the specific detection of that SE subtype [16]. Also, secretion of the cytokine TNF (tumor necrosis factor) by murine primary cells has been applied to the detection of SEA [17].

In this study, we harnessed the superantigenic effect of SEA and used an ex vivo bioassay as well as a cell line-based assay for SEA detection. Both these bioassays can differentiate between active and inactive SEA. The ex vivo method has advantages over the in vivo monkey and kitten bioassay because one mouse spleen can provide enough cells for roughly 500 assays. However, this ex vivo bioassay requires sacrificing of mice. To deal with the limitations of this ex vivo bioassay we examined CCRF-CEM, a human CD4^+^ T-cell line and developed an in vitro cell based assay for the detection of active SEA as an alternative to the current animal testing. 

## 2. Results

### 2.1. IL-2 Secretion Assay for Measuring Biologically Active SEA in Food

**Figure 1:**
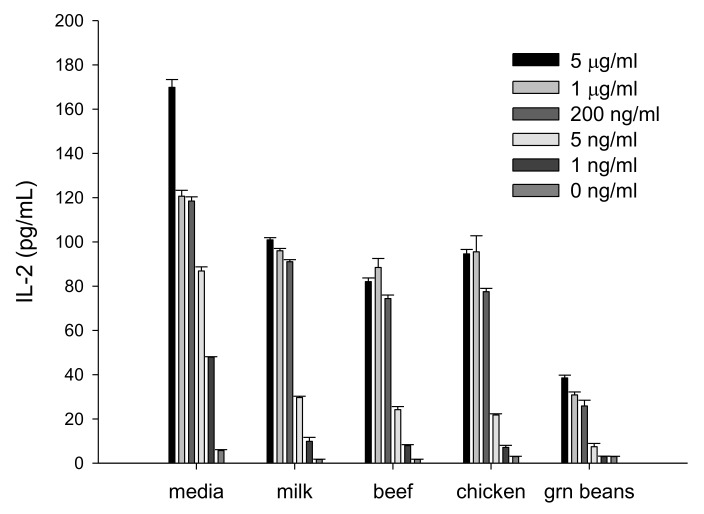
Interleukin-2 (IL-2) is a quantifiable measurement for detecting biologically active staphylococcal enterotoxin A (SEA) in various food matrices. Different food matrices were spiked with various concentrations of SEA and incubated for 24 h. Immunomagnetic beads were used to isolate SEA. The isolated toxin was incubated with splenocytes. IL-2 secretion was measured by ELISA assay. Error bars represent standard deviation. µg = microgram; ng = nanogram.

We evaluated if IL-2 secretion by activated splenocyte cells can be used for detection of SEA in different food products. Milk, beef, chicken, and green beans were spiked with various concentrations of SEA and samples were incubated with splenocyte cells. However, due to food matrix interference, the high background reduced the signal-to-noise ratio. To eliminate this matrix interference, we separated SEA from spiked food products by employing immunomagnetic beads that were coated with an anti-SEA antibody. The toxin was extracted from beads and incubated with mouse spleen cells for 24 h. In Figure 1 our ELISA shows that we can detect SEA in food products and that there is a correlation between the extracted SEA concentrations and IL-2 protein secretion from splenocyte cells.

### 2.2. Quantitative In Vitro T-cell-Based Assay for Quantifing Biologically Active SEA

**Figure 2:**
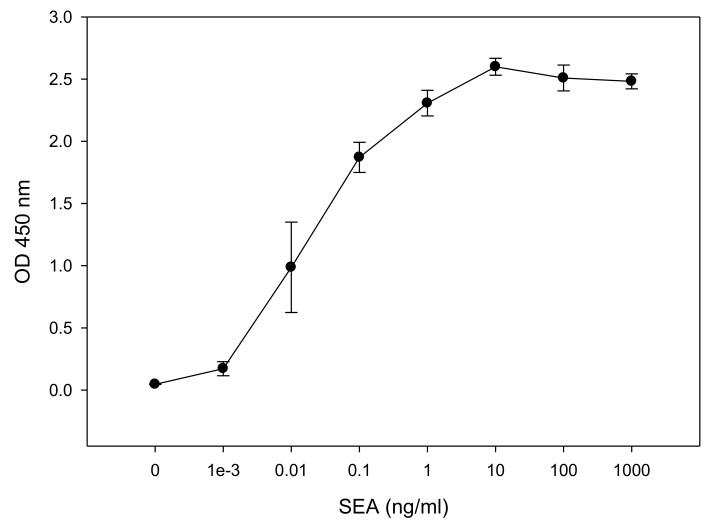
In vitro assay for measurement of biologically active SEA. CCRF-CEM T-cells and Raji B-cells were mixed in a co-culture and were incubated for 48 h with increasing concentrations of SEA. Induced IL-2 secretion was measured in dose dependent response. Error bars represent standard errors. OD (Optical Density).

This ex vivo method has further advantages over the in vivo monkey and kitten bioassays. As well as dramatically limiting the number of animals used, one mouse spleen can be used for 500 tests, such an ex vivo bioassay requires the sacrifice of living animals and therefore raises the same ethical concerns regarding the use of experimental animals. Accordingly, we sought an alternative to mouse splenocytes and so performed experiments to evaluate the use of the human CD4^+^ T-cell line CCRF-CEM for the measurement of biologically active SEA. This was done in conjunction with Raji B-cells as antigen presenting cells (APCs). After incubation for 24 h of the co-culture with various concentrations of SEA, ELISA was used to quantify the relative concentration of secreted IL-2. The ELISA results in Figure 2 show that SEA induces differential IL-2 protein secretion in a dose-dependent manner. One-way analysis of variance (ANOVA) was applied to determine the limit of SEA detection. The result shows that the sensitivity of the assay for SEA is 0.01 ng/mL. Figure 2 shows that 0.010 ng/mL SEA increases ELISA detection of IL-2 secretion from 0.0463 ± 0.00145 OD (Optical Density) in the control case to 0.987 ± 0.363 OD with a *p*-value < 0.001 (lower than 0.05).

### 2.3. Distinguishing Active from Inactive SEA

**Figure 3:**
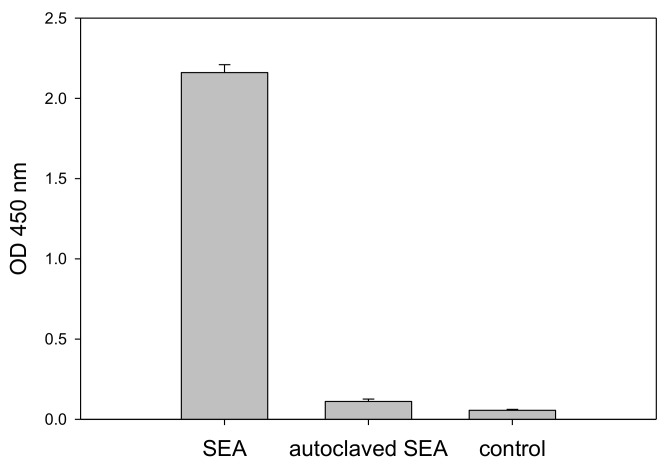
Assay discrimination of biologically active from inactivated SEA. CCRF-CEM T-cells and Raji B-cells, in a mixed culture, were incubated for 48 h with 1 µg/mL SEA, thermally treated SEA, and a phosphate-buffered saline (PBS) control. Secretion of IL-2 was measured by ELISA. Error bars represent standard errors.

It is important to be able to distinguish active from inactive toxin. Thermal processing is the primary standard by which foods are treated for the elimination of bacterial contamination as well as to inactivate their toxins. We next examined the response to heat inactivated SEA. SEA was heated by autoclaving for 15 min (15 psi, 121 °C) and compared with unautoclaved, active SEA in the CCRF-CEM T-cell line assay. Figure 3 shows that only active SEA is able to induce IL-2 secretion during the incubation period, while heat inactivated SEA produces results comparable to the control without SEA. This result demonstrates that the assay can distinguish between biologically active SEA and heat inactivated SEA which poses no health risks. 

### 2.4. Cross-Reactivity Assay Specificity and Function of CCRF-CEM Cells Elicited by SEA

**Figure 4:**
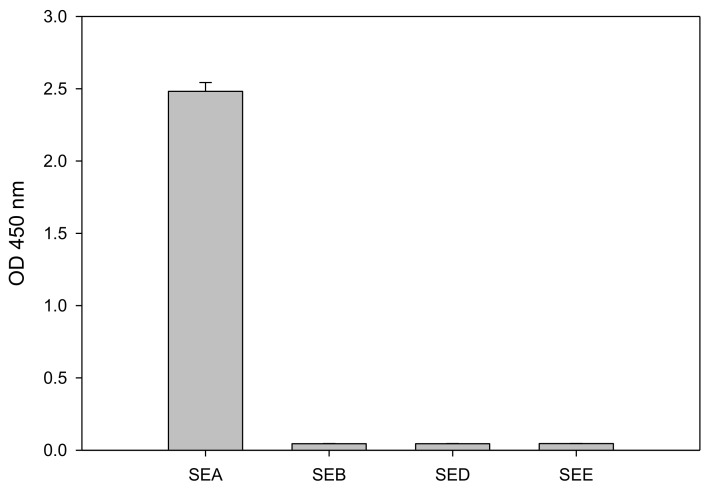
ELISA assay specificity as determined by secretion of IL-2. CCRF-CEM T-cells were co-incubated with Raji B-cells and SE types A, B, D or E (SEA, SEB, SED, and SEE, respectively) for 48 h. Error bars represent standard errors.

SEs shares considerable amino acid sequence similarity and therefore the specificity and cross reactivity of the assay for SEA should be evaluated. To inspect the specificity of the test we applied 1 µg/mL of SEA, SE types B, D and E (SEB, SED, and SEE, respectively) to the CCRF-CEM T-cell line in combination with the Raji B-cell line. As shown in Figure 4, SEA but not SEB, SED, or SEE stimulate the CCRF-CEM T-cell activation, as demonstrated by IL-2 secretion. Although SEA and SEE share 70–90% sequence homology [18] and consequently belong to the same SE group [19,20], SEE produces no response. Only SEA is able to activate the CCRF-CEM T-cell and induce secretion of IL-2. This result indicates that this test is specific to SEA detection. 

### 2.5. Detection of SEA in Whole Milk

**Figure 5:**
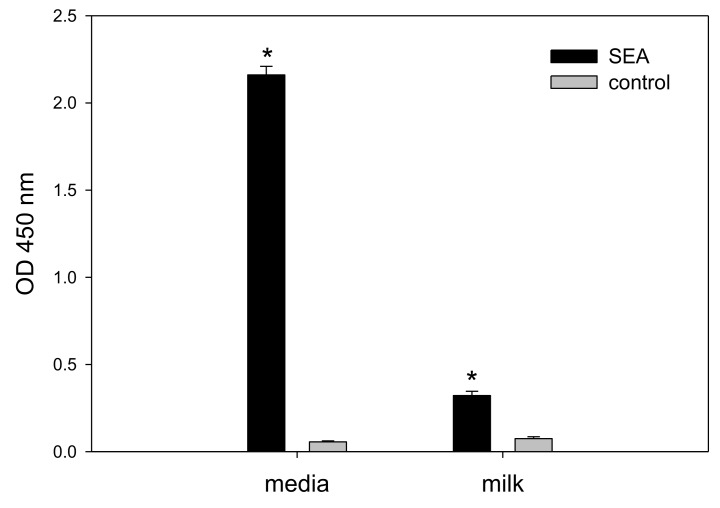
Detecting 1 µg/mL of SEA in spiked milk. Spiked milk was added to 85 µL of cell culture media; IL-2 was analyzed after incubation for 24 h. Error bars represent standard errors, and significant differences (*p* < 0.05) between spiked and un-spiked milk are represent with an asterisk*.

The importance of detecting active SEA and preventing it from entering the human food chain is highlighted by an outbreak in contaminated milk in Japan causing 13,420 cases of intoxication [5,6]. Therefore, we investigated the food matrix effect of milk on the cytokine secretion assay. Whole milk was spiked with SEA and assayed. Our result in Figure 5 shows that whole milk interferes with and reduces cytokine secretion and consequently reduces the signal-to-noise ratio. However, IL-2 secretion in SEA spiked whole milk was statistically significant compared to the un-spiked whole milk control. A t-test analysis between spiked and un-spiked milk samples shows that milk spiked with 1 µg/mL SEA increases ELISA detection of IL-2 secretion from 0.075 ± 0.0104 OD to 0.3220 ± 0.0243 OD with a *p*-value < 0.001 (lower than 0.05).

## 3. Discussion

In this current study we developed an activity assay as an alternative to the current live animal bioassays, for the detection of biologically active SEA. The type A variant of Staphylococcal enterotoxin is associated with (78%) of staphylococcal outbreaks in the US [8]. The existing method to detect SEA is to administer SEA directly into the stomach of monkeys or kittens and then observe for any emetic response [9,10]. This method of detection is expensive, difficult to reproduce, and has low sensitivity. For an administration of 10 µg SEA, vomiting occurs with 50% of the animals [9,10,21]. An immunological assay that was developed for food safety can measure the presence of SEA but cannot differentiate between the active form of SEA, which is a threat to public health, and inactive SEA [12]. The ability to discern active toxin is also important for development of food treatment and processing methods. Cooking and pasteurization are forms of heat treatment that inactivate SEA [22]. Pulsed ultraviolet (UV) light has also been applied to inactivate SEA [23].

Previous work has shown that there is a relationship between the emetic activity of SEs and their superantigenic activity. When site-directed mutagenesis was used, it inhibited SEC2’s (Staphylococcal Enterotoxin Type C2) emetic activity, resulting in elimination of its superantigenic activities [4]. Therefore, it was hypothesized that an assay that can quantify superantigenic activities can be used to predict emetic activities. Bavari et al. and Hufnagle et al. utilized human peripheral blood mononuclear cells (PBMC) and measured T-cell proliferation using 3H thymidine incorporation [24,25]. However, using PBMCs from healthy human donors adds complexity and they are not always available. In addition, this method produces radioactive waste and is therefore not a desirable alternative method to the in vivo bioassays for detection of active SEA. We applied a different approach in this study by replacing PBMCs with mouse splenocytes and replaced the quantitative detection of radioisotope 3H thymidine with IL-2 secretion by activated CD4^+^ T-cells. As demonstrated by Figure 1, IL-2 represents an early molecular response to SEA. Early detection of IL-2 secretion can be quantified in less than 24 h. We observed that the food matrices interfere with the assay. The food matrix interference reduces the signal-to-noise ratio, and was removed by employing immunomagnetic beads coated with an anti-SEA antibody to isolate the SEA from the spiked food. SEA was eluted from the beads and added to the cells, inducing IL-2 secretion in a dose-dependent manner (Figure 1). This ex vivo bioassay produces no radioactive waste and requires no radioisotopes as in the PBMC assay, making it a more easily practical and available method for laboratory use. 

The Lautenberg Chemical Safety Act encourages the development and adoption of alternatives to testing on animals for chemical toxicity methodologies. Even though this IL-2 secretion bioassay dramatically reduces the number of animals used, i.e., a single mouse spleen can be used for 500 tests, there is still the requirement to sacrifice live animals in order to obtain the requisite splenocytes. Thus, this approach raises the same ethical concerns regarding the use of experimental animals. We took a step further to eliminate this concern by replacing the mouse splenocytes with a human cell line to avoid all animal testing. The data presented here show, for the first time, the successful implementation of an activity assay for SEA using a CD4^+^ T-cell line in combination with a B-cell line which presents the SEA-MHC class II complex to the CD4^+^ T-cell line. This in vitro assay showed that increasing concentrations of SEA induced a dose dependent IL-2 secretion in the range 0.001 ng/mL to 10 ng/mL SEA (Figure 2). The data presented here show that the detection limit of this IL-2 secretion assay is 100 times higher than the p50 of SEA reported for human PBMCs [26] and is equal to the detection limit of 10 pg/mL reported by Rajkovic et al. for their highly sensitive immune-PCR assay, which detects both active and inactive SEA [27]. 

In humans, the estimated 50% lethal dose (LD50) is 0.02 μg/kg and the 50% effective dose (ED50) is 0.0004 μg/kg by aerosolized exposure [28]. At a concentration of 500 pg/mL of SEA in milk, nearly one third of those consuming a 280 mL carton in a Japanese outbreak were sickened and it was estimated that between 20 and 100 ng of SE in food is sufficient to cause food poisoning [5,29]. At a level of detection of 10 pg/mL, our assay is 50-fold more sensitive than required in this case. Compared with a latex agglutination assay for SEA having a limit of detection of 0.5 ng/mL, our method is 500 times more sensitive while also having the advantage to respond only to active toxin [12].

Assay specificity is critical for food safety analysis and so we evaluated the assay specificity for (1) inactive toxin, (2) cross activity with other SEs, and (3) the background signal in milk. The specificity analysis with very high concentration of heat inactivated SEA (1 µg/mL) showed no response (Figure 3) suggesting that the assay is very specific to active SEA which does induce secretion of IL-2. Moreover, the assay is specific to SEA with no cross reactivity with other common SEs including SEB, SED, and SEE (Figure 4) despite the fact that SEs have considerable amino acid sequence similarity. SEA and SEE share a 70–90% sequence homology and consequently belong to the same group of SEs [19,20]. SEE did not mediate secretion of IL-2 or any T-cell response. It is possible that the observed difference in T-cell responses between SEA and SEE is the binding specificities to the TCR (T-cell receptor) β chain protein expressed on the CCRF-CEM T-cell line. When a Jurkat T-cell line was used in a similar cell-based assay for SEE, that assay was very specific to SEE with no cross reactivity to SEA [14,15]. SEA food poisoning is often associated with milk, but whole milk interferes with and reduces cytokine secretion and significantly reduced SEA detection (Figure 5). This suggests that for milk there is a need for purification of the toxin (e.g., using magnetic beads) prior to the assay. The new in vitro assay is simple, rapid, and an inexpensive alternative to the in vivo bioassay.

## 4. Materials and Methods

### 4.1. Media and Reagents

All media components were sources from Gibco (Gibco/Thermo Fisher, Waltham, MA, USA) unless otherwise noted. Cell culture medium was comprised of RPMI 1640 with the addition of 10% fetal bovine serum (HyClone, Logan, UT), 200 mM glutamine, 1 mM sodium pyruvate, 1× MEM (Roswell Park Memorial Institute medium )nonessential amino acids, and antibiotic-antimycotic. An addition of 50 μM β-mercaptoethanol (Sigma, St. Louis, MO, USA) was added to media for murine cells. Lysis buffer was comprised of 150 mM NH_4_Cl, 10 mM KHCO_3_, and 100 μM Na_2_EDTA. SEA, SEB, SED, and SEE toxins were obtained from Toxin Technology (Sarasota, FL, USA). All toxins have a >95% purity level obtained from SDS-PAGE and Coomassie blue stain. The toxins were reconstituted in water. Anti-SEA antibody was obtained from Toxin Technology (Sarasota, FL, USA). Human IL-2 ELISA assay (Cat 555190) was obtained from BD Bioscience (San Diego, CA, USA).

### 4.2. Cells and Cell Lines

The human T lymphoblastoid line CCRF-CEM cells (ATCC CCL-119 (American Type Culture Collection catalog number CCL-119)), an isolate from the peripheral blood of a 4-year old Caucasian female with acute lymphoblastoid leukaemia, were obtained from the American Type Culture Collection (Rockville, MD, USA). Raji cells (ATCC CCL-86), a human Burkitt’s lymphoma B-cell line, were obtained from the American Type Culture Collection (Rockville, MD, USA). Both CCRF-CEM and Raji cells were maintained in cell culture medium. Murine splenocytes were isolated from spleens aseptically as previously described [14]. All cells were maintained in a 37 °C incubator under a humidified 5% CO_2_ atmosphere.

### 4.3. Methods of Preparation of Foods for Analysis

Twenty grams of preserved beef paste, chicken paste, or green beans, (Gerber, Fremont, MI, USA) were added to 20 mL of phosphate-buffered saline (PBS) and spiked with SEA. Milk was prepared by adding nonfat dry milk and water for a final concentration of 5% (Nestle, Solon, OH, USA). Whole milk was obtained from a local market.

### 4.4. SEA Magnetic Beads Preparation

Tosylactivated Dynabeads M-280 (100 μL) (Invitrogen, Carlsbad, CA, USA) were washed two times with 600 μL of 0.1 M sodium borate buffer, pH 9.5. Then they were diluted to 2 × 10^9^ beads/mL in the same buffer. Anti-SEA purified antibody (30 μg) was added to 1 × 10^8^ beads (50 μL). To facilitate covalent binding the beads and antibody were incubated at 37 °C for 24 h on a slow shaker. The coated beads were then washed twice with 1 mL PBS, pH 7.4, containing 0.1% bovine serum albumin (BSA), for 5 min at 4 °C, then incubated for 4 h at 37 °C with 0.2 M Tris-HCl, pH 8.5, containing 0.1% BSA. The final wash was with PBS, pH 7.4, containing 0.1% BSA for 5 min at 4 °C. The beads were resuspended in Tris-BSA buffer (50 μL).

### 4.5. Sample Binding and Disassociation of SEA from Beads

Immunomagnetic beads (15 µL) were incubated with a tilting motion at 4 °C with 4 mL of food spiked with SEA for 24 h. The beads and spiked food were placed on a magnet for 2 min to collect the beads. The beads were washed twice with PBS, pH 7.4, containing 0.1% BSA. Toxin was eluted with 7.5 μL of 100 mM glycine-HCl (pH 2.5) then neutralized with 7.5 μL of 2× Tris-buffered saline (TBS) (pH 8.3).

### 4.6. Quantitative Determinations of Active SEA by IL-2 Protein Secretion

In a clear 96-well plate, 50 µL of 2 × 10^6^ cells per mL suspension of CCRF-CEM cells, 25 µL of a 2 × 10^6^ cells per mL suspension of Raji cells, and 25 µL of SEA at four times final target concentration were combined in cell culture medium. Cells were then incubated at 37 °C for up to two days. The appropriate controls were: treatment without SEA represented a negative control and a positive control was any SEA-containing sample. Supernatants were harvested after 24 and 48 h and were tested for IL-2 by ELISA following the manufacturer’s instructions (BD OptEIA Human IL-2 ELISA). For the purpose of comparison the ELISA results are left in terms of OD 450 instead of conversion to concentrations which requires freshly prepared IL-2 standards.

### 4.7. Statistical Analysis

Statistical analysis was performed with SigmaStat 3.5 for Windows (Systat Software, San Jose, CA, USA). One-way analysis of variance was used to determine the detection of SEA. The experiments were repeated at least three times, and results with *p* < 0.05 were considered statistically significant. A *t*-test analysis was used to determine whether there were any significant differences between treatment and control.

### 4.8. Ethics Statement

Procedures with animals were performed in accordance with institutional guidelines for husbandry, which have been approved by the Institutional Animal Care and Use Committee of the U.S. Department of Agriculture, Western Regional Research Center. Mice were euthanized by rapid cervical dislocation to minimize suffering, following protocol no. 13–15 January 2016.
